# Matters of the Heart: Co-Creating a Peer-Led Social Health Intervention for People Living with Dementia

**DOI:** 10.3390/bs16010009

**Published:** 2025-12-20

**Authors:** Doris Gebhard, Leonie Lang

**Affiliations:** 1 School of Medicine and Health, Technical University of Munich, 80809 Munich, Germany; leonie.lang@ebw-muenchen.de; 2 Protestant Educational Institute Munich e.V., 80331 Munich, Germany

**Keywords:** dementia, social health, long-term care, co-creation, intervention, participation, peer-approach, empowerment

## Abstract

Social health is increasingly recognized as a key domain in dementia research, yet interventions explicitly addressing it remain scarce. This study presents the co-creation of an empowering and meaningful social health intervention for people living with dementia. An evidence-based intervention scaffolding was enriched with the lived experiences of people living with dementia through a seven-step co-creation process, in which they held sole decision-making authority in selecting intervention topics using an adapted World Café method, shared responsibility for designing session content, and joint responsibility for implementation. Twenty-nine residents living with dementia in three long-term care facilities co-created and implemented twelve group sessions based on their “heart topics,” emphasizing personal strengths, reciprocity, and shared experiences. Each session integrated peer-led, co-creative, and sensory elements and was collaboratively prepared and implemented together with at least one peer host. The co-creation process effectively captured the lived experiences of people living with dementia and resulted in an intervention with the potential to foster and deepen social relationships in long-term care. This study calls on researchers and practitioners to take bolder steps toward empowering people living with dementia to assume active, visible, and meaningful roles in intervention development and implementation.

## 1. Introduction

Dementia is a syndrome characterized by a decline in cognitive function that significantly affects an individual’s physical, mental, and social health, leading to a progressive loss of independence and quality of life (Gale et al., 2018). It is an umbrella term encompassing several diseases with different causes and manifestations, most commonly Alzheimer’s disease, but also, for example, vascular dementia, dementia with Lewy bodies, Parkinson’s disease dementia, and frontotemporal dementia (World Health Organization, 2017). Dementia is not limited to cognitive decline; it is also associated with behavioural and psychological symptoms, including changes in behaviour, perception, thought content, and mood (Jönsson et al., 2025). These symptoms occur across dementia types but may present differently and have varying impacts on the lives of people living with dementia, including their social relationships (Jönsson et al., 2025; Schwertner et al., 2022). Regardless of type, dementia may alter the social lives of those affected due to at least three key factors (Alzheimer’s Disease International, 2024; Boamah et al., 2021; Schwertner et al., 2022): (1) Cognitive impairments and behavioural symptoms make it more difficult to maintain relationships, engage in social activities, or participate in conversations. (2) Social environments change as dementia progresses—particularly with relocation to long-term care—reducing opportunities for contact with loved ones. (3) Dementia-related stigma may lead to withdrawal, discrimination, and fewer social opportunities.

In recent years, the *social* component of *psychosocial* health in people living with dementia has gained increasing attention. Once viewed mainly as an adjunct to psychological health, it is now recognized as an equally important domain within a holistic bio-psycho-social understanding of health (Doyle & Link, 2024; Spector & Orrell, 2010). This shift has been strengthened by the dementia-specific operationalization of social health (Dröes et al., 2017) and further accelerated by the INTERDEM Manifesto in 2019, which highlighted social health as a priority in dementia research (Vernooij-Dassen et al., 2021). Subsequent progress includes a conceptual framework of social health in dementia (Vernooij-Dassen et al., 2022), an overview of relevant social health markers (Kristanti et al., 2024), and a review of measures to assess social health in people living with dementia (Altona et al., 2024). These developments lay the foundation for interventions that specifically address social health in this population.

### 1.1. Informing Social Health Intervention Development

Existing evidence on psychosocial interventions for people living with dementia provides only limited guidance on how to address social health. The umbrella term of psychosocial interventions covers a wide range of effective non-pharmacological approaches for people living with dementia (Luxton et al., 2026), with cognitive-behavioral interventions, cognitive rehabilitation, stimulation therapy, music therapy, physical activity, reminiscence, and sensory stimulation being most recommended in European dementia care guidelines (Neal et al., 2025). While most psychosocial interventions include both psychological and social components, these are often not clearly differentiated. Consequently, it remains difficult to identify the underlying mechanisms of change, as the specific contributions of individual components have rarely been analysed (McDermott et al., 2019). Although the specific components of interventions (the *what*) cannot be directly transferred with certainty regarding their impact on social health, effective approaches to intervention design (the *how*) offer valuable guidance for developing social health interventions. Among these, tailoring interventions to participants’ characteristics, preferences, interests, and needs has been identified as a key mechanism for both effectiveness and feasibility of interventions in people living with dementia (Durgante et al., 2025; Tournier et al., 2023). Thus, interventions to address social health should be designed in ways that facilitate participation according to individual preferences, competencies and meaningfulness. This perspective is rooted in the philosophy of person-centered dementia care, which places the unique needs, values, goals, preferences, and subjective experiences of people living with dementia at the center (Fazio et al., 2018). Accordingly, van Haitsma et al.’s (2020) Preference-Based Model of Care offers a suitable theoretical basis for developing interventions that address social health in this population. The model draws, among four other theories, on self-determination theory (Ryan & Deci, 2000), which provides a particularly relevant foundation for our approach, as it identifies autonomy, competence, and relatedness as the three basic psychological needs closely linked to motivation and well-being (Ryan & Deci, 2017).

#### Individual Competencies and Meaningfulness

To enable participation across the spectrum of dementia severity, tailoring interventions to the implementation environment, timing, complexity, and level of support has proven effective (Regier et al., 2017; Wyman et al., 2022). However, existing tailoring strategies focus mainly on compensating for dementia-related losses, with far less attention to approaches that build on individual strengths and competence (Jackman et al., 2024). An empowering perspective shifts the focus from what is lost to what remains possible. To support such intervention development, van Corven et al. (2021) proposed a conceptual framework of empowerment for older people living with dementia, identifying four key themes: (1) maintaining personal identity, (2) experiencing choice and control, (3) feeling useful and needed, and (4) retaining a sense of worth. When asked directly, people living with dementia identified “heart oriented” strengths such as love, kindness, and humor as most important for living well (Jackman et al., 2024). Therefore, designing interventions that create social contexts enabling these strengths to be expressed and practiced can be recommended.

The question of what makes activities meaningful for people living with dementia has been examined in the literature across different layers. Studies on specific meaningful activities for people living with dementia report a wide range, from everyday routines such as reading the newspaper, watching television, shopping, and cooking together to physical exercise, nature experiences, and even advocacy work (Tournier et al., 2023; Tuijt et al., 2020). Beyond specific activities, Tierney and Beattie (2020) proposed a conceptual model defining five attributes of meaningful activity for older adults living with dementia: it should be enjoyable, identity related, engaging, goal oriented, and individually tailored. While this model provides a useful conceptual basis, the authors note that practical guidance on translating these attributes into interventions remains limited. Extending these perspectives, A. Han et al. (2016) synthesized research on the lived experiences of people living with dementia and identified a unifying mechanism underlying meaningful activities: fostering a sense of connection. This connection unfolds across three dimensions: (1) with oneself, through activities that support identity and health; (2) with others, through activities that promote belonging; and (3) with the environment, through activities that sustain a relationship with the physical world.

### 1.2. Development of the “Matters of the Heart” Social Health Intervention

The outlined theoretical foundation and literature on individual competencies and meaningfulness provide important insights for designing social health interventions. Building on this, we first designed an intervention scaffolding. Second, we brought this conceptual foundation to life with content grounded in the lived experiences of people living with dementia using a co-creation approach, which is the core of the present study.

#### 1.2.1. Designing the Intervention Scaffolding

The intervention scaffolding is structured around the three dimensions of feeling connected (A. Han et al., 2016). For each dimension, we selected an intervention approach to best address the respective level of connectedness:

- Enhancing Connection with Oneself

Peer-led formats offer a promising way to strengthen individuals’ connection with themselves (Neuhaus et al., 2022). In these approaches, people living with dementia assume leadership roles, shifting from passive recipients to active facilitators and gaining opportunities to express their talents and capabilities (Theurer et al., 2015). Although peer support interventions are increasingly common in community settings (e.g., Miyamae et al., 2023; Sullivan et al., 2022), peer-led interventions remain rare. A pioneering study nevertheless showed that people living with dementia in residential long-term care can successfully lead diverse group activities for their peers (Skrajner et al., 2014).

- Enhancing Connection with Others

This dimension aligns with a central marker of social health: *social connectedness*, defined as having meaningful, close, and constructive relationships (Liougas et al., 2024; O’Rourke & Sidani, 2017). Activities that foster this sense of connection can therefore guide social health intervention design. One way of translating this principle into practice is through *co-creativity*, characterized by shared process, shared ownership, inclusivity, reciprocity, and relationality (Zeilig et al., 2018). For people living with dementia, co-creativity supports relational interactions grounded in shared, playful experiences rather than instrumental tasks. An illustrative example is the *With All* project, where co-creative arts activities enabled joint creative engagement, offering opportunities for communication, expression, and doing things together (Zeilig et al., 2019).

- Enhancing Connection with the Environment

Perceiving the environment through all senses, and thereby fostering a sense of connection with it, can be supported through sensory stimulation (Hayden et al., 2022). This approach creates pleasurable experiences that engage the primary senses without requiring intellectual effort (Yang et al., 2021). Because it does not rely on cognitive abilities, it is well suited for people in advanced stages of dementia, whose opportunities for verbal communication are limited. In our intervention, we use olfactory (O), visual (V), auditory (A), tactile (T), and gustatory (G) stimuli to nurture connectedness.

#### 1.2.2. Co-Creating the Intervention Content

According to Vargas et al. (2022), co-creation is an overarching concept that integrates both co-design and co-production. In their six-step *Model for Co-Creation of Public Health Initiatives*, co-design comprises identifying, analysing, defining, and designing, whereas co-production involves realising and evaluating. Because this study includes both the design and implementation of a social health intervention, we use the broader term co-creation. Tsekleves et al. (2020) also argue that co-creation is the most appropriate term for participatory research with people living with dementia, further supporting our terminology. Despite blurred boundaries between related concepts, all co-methods share participatory principles centred on agency and voice (Niner et al., 2023). However, co-creation offers opportunities for meaningful participation, shared ownership, and relationship-building, fostering a sense of connection and integration. By amplifying participants’ voices and redistributing decision-making power, it enhances empowerment and positions people living with dementia as active agents rather than passive contributors (Agnello et al., 2025). Involving people living with dementia as experts by lived experience is now regarded as ethical best practice and has demonstrated benefits both for participants and for the design process (Wang et al., 2019). Building on this, our study engages people living with dementia as active partners in designing and implementing the intervention. Recent reviews also highlight the need for greater transparency in reporting co-creation procedures to support replication and adaptation of effective approaches (Agnello et al., 2025). By providing transparency about both methods and outcomes, future interventions can build on our experiences and advance participatory dementia research.

### 1.3. Study Aim

The aim of this study is to present both the detailed steps and key outputs of the co-creation process of intervention development and implementation, with the dual purpose of offering a transferable process model for future interventions and introducing a social health intervention that can be tailored to individual themes and applied in practice.

## 2. Materials and Methods

### 2.1. Study Design

This study is part of a larger research project (“CaResource”) focusing on the everyday lives and health of people living with dementia in long-term care facilities. The project follows a multiperspective, merged methods approach and aims to ensure a high degree of participation and empowerment of people living with dementia throughout all phases of the research. As part of the project, a comprehensive needs assessment was conducted (Gebhard et al., 2024; Gebhard & Frank, 2024), forming the basis for the development, implementation, and evaluation of an intervention addressing the physical, psychological, and social health of residents with dementia. This article focuses specifically on the development process of the intervention component targeting social health. The study was approved by the Ethics Committee of the Technical University of Munich (357/21 S) and registered with the German Clinical Trials Register and the WHO International Clinical Trials Registry Platform (DRKS00029555).

### 2.2. Setting and Sample

Three of the six residential long-term care facilities involved in the overall project participated in the intervention development and implementation. The participating facilities were traditional nursing homes, all located in the city of Munich (Bavaria, southern Germany), with sizes ranging from 133 to 208 residents (mean = 172.0, standard deviation = 37.59). In each facility, a convenience sample of residents was recruited in consultation with the care manager, based on the following inclusion criteria: (1) a documented diagnosis of dementia in the care records, including all dementia types (in most cases a differential diagnosis was not available) but excluding mild cognitive impairment, (2) being 65 years of age or older at the time of inclusion, as the study did not focus on people with young onset dementia (defined as being under 65 years of age), who typically require different intervention approaches, particularly in the psychosocial domain (Mack et al., 2025); and (3) not being cared for in bed, as the social health intervention was, in the further course of the project, combined with a physical activity program that required a minimum level of mobility (at least the ability to be mobile with the support of a wheelchair) (Gebhard & Mess, 2022; Gebhard & Ellinger, 2025). The same individuals were involved in both the development and implementation of the intervention. The research team approached the pre-selected potential participants in face-to-face situations, explained the aims of the study, the procedures, and their personal motivation for conducting the research. Written informed consent was obtained from all people living with dementia who decided to participate and, where applicable, from their legal representatives. The right to withdraw from the study at any time was fully respected, including instances of situational dissent. The intervention development and implementation took place between May and October 2021. 

Age, gender, and care level were extracted from participants’ care records. The care level refers to a five-level German system that indicates the degree of support a person requires for daily activities (Federal Ministry of Health, 2025); higher levels reflect greater dependency. Cognitive functioning was assessed using the Mini-Mental State Examination (MMSE; Folstein et al., 1975; Mitchell, 2009), a 30-item screening tool with a maximum score of 30 points, where lower scores reflect more severe cognitive impairment. The planned assessment of behavioural and psychological symptoms of dementia using the Neuropsychiatric Inventory (Reuther et al., 2016) could not be carried out, as care staff were unable to complete the proxy instrument due to insufficient time resources.

### 2.3. Co-Creation Process

**Figure 1:**
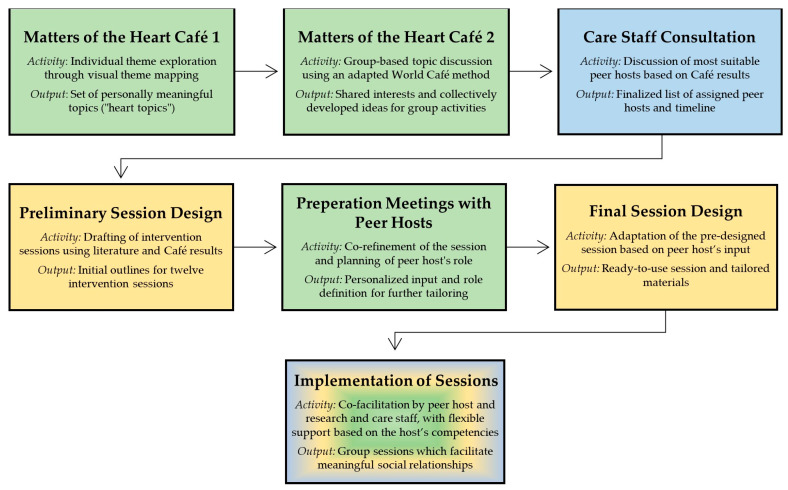
The seven steps of the co-creation process.

The applied co-creation process consisted of seven sequential steps. Figure 1 illustrates the process, presenting the steps with their key activity and output. The different colours indicate the main group of actors carrying out each activity and holding decision-making power: green = people living with dementia, yellow = research team, and blue = care staff. 

The co-creation process was designed in accordance with recommendations for co-designing with people living with dementia defined in the review by Wang et al. (2019). First, in line with recommendations for location, co-creation sessions were conducted in familiar environments: group sessions took place in the communal room or garden of the care facility, while individual sessions were held in the participant’s preferred setting (private room, communal room, or garden). Second, the research team met recommended criteria for working with people living with dementia: being flexible, empathetic, patient, and well-informed about residents’ daily lives. All components of the co-creation process, including data analysis, were conducted by the two female authors. Both have extensive experience in long-term care. One holds a PhD in Health Promotion and is employed as a postdoctoral researcher at the university; the second author held a Bachelor’s degree in Health Sciences at the time and was completing a Master’s program in Gerontology alongside her position as a research assistant. Both were trained in qualitative and participatory research. Both researchers were already familiar to participants and had established strong relationships with staff through extensive on-site presence during the project’s needs assessment phase. The two researchers were supported on specific tasks by additional research assistants, all of whom held at least a bachelor’s degree in health sciences, worked under supervision, and had received prior training. Third, following the recommendation on the structure, we organized the group sessions as three parallel small groups of three to four participants, each facilitated by a researcher or care staff member. Breaks were included flexibly and informally in response to participants’ needs. Fourth, involvement methods were deliberately chosen to match the objectives of each stage in the process and adapted to the needs and abilities of individual participants. Regardless of the specific method, each session began with a clear explanation of the overall purpose of the co-creation process, the specific aims of the session, and reassurance that all contributions—whether sharing stories, expressing opinions, or simply listening—were valuable. 

### 2.4. Data Generation and Analysis in the Co-Creation Process

Throughout the co-creation process, different types of data and information were generated, which were documented, analysed, and used in various ways depending on their purpose. 

The Matters of the Heart Cafés 1 and 2 produced qualitative data that were systematically analysed. Following Café 1, transcripts were created for each participant based on the individual portrait boards and accompanying field notes. The transcripts were analysed using qualitative content analysis (Mayring, 2022). Inductive categories of heart topics were first generated through summarization. The material within these topics was then structured along the three deductive dimensions of *Me for Myself*, *Me for the Group*, and *The Group for Me*, and subsequently summarized. All transcripts were analysed independently by two coders. To ensure reliability, discrepancies between coders were resolved through discussion and consensus. Qualitative data analysis was conducted using MAXQDA 2024. Following Café 2, transcripts were created for each heart topic based on the generated topic sheets and accompanying field notes. These transcripts were collaboratively synthesized into narrative summaries by the two coders, integrating individual contributions, group discussions, and field notes into coherent accounts. Participating people living with dementia were not involved in data analysis or interpretation. The information collected during the care staff consultation and preparation meetings with peer hosts was documented but not analysed, as these data served to inform subsequent process steps within each care facility and to individualize the session procedures. The descriptions of the developed sessions, arising from the intermediate step *Preliminary Session Design* and the subsequent *Final Session Design*, were recorded in implementation plans. Accordingly, the following results section presents the procedures across all steps of the co-creation process, reports the findings from the first two steps (Matters of the Heart Cafés 1 and 2), and provides an insight into the resulting intervention sessions.

During the preparation of this manuscript, the authors used ChatGPT-5 (OpenAI) for the refinement of English language and grammar. The authors have reviewed and edited the output and take full responsibility for the content of this publication

## 3. Results

### 3.1. Sample 

In total, 37 people living with dementia were invited to participate in the study, and 31 provided informed consent. Of these, one person declined participation during the intervention development process (situational dissent) due to visual and hearing impairments, and one participant passed away. Thus, a total of 29 people living with dementia participated in the intervention development (Matters of the Heart Café 1 and 2; preparation meetings with the peer hosts) and the implementation of the developed intervention (10 each in facilities 1 and 2, and 9 in facility 3); two of them were male. Table 1 presents the characteristics of the participants.

### 3.2. Matters of the Heart Café 1

#### 3.2.1. Implementation

The first Matters of the Heart Café was conducted as a 90 min group session. In a relaxed café-style setting, small groups of three to four residents sat at decorated tables with coffee and cake provided. Each participant had a personalized portrait board with their name and three color-coded cards representing the themes for discussion (see Table 2 for themes and questions). Using these visual prompts, research staff engaged participants in brief individual conversations within the group setting, asking the related questions and documenting each response on small cards. These were attached to the corresponding theme cards on the portrait boards to visually map the participants’ “heart topics”. Additionally, field notes were taken. Commonalities and individual preferences were summarized at each table and shared across tables at the end of the session. 

#### 3.2.2. Findings

The analysis of the first Matters of the Heart Café identified twelve heart topics in participants’ narratives. The identified heart topics and a category of general statements were summarized across the three themes Me for Myself, Me for the Group, and The Group for Me and are presented in Table 3.

### 3.3. Matters of the Heart Café 2

#### 3.3.1. Implementation

Like the first, the second Matters of the Heart Café took place in a relaxed café-style setting and lasted around 90 min. The procedure was based on the principles of the World Café method (Löhr et al., 2020) but adapted for the context of long-term care and the needs of people living with dementia. In contrast to the original World Café approach, participants remained seated while the table hosts—members of the research team—rotated between tables, bringing along a visual topic sheet for each discussion round. Each sheet (A3 format) displayed one of the heart topics identified during the first Matters of the Heart Café, along with a thematically related photograph serving as a visual storytelling prompt. In addition, relevant contributions from the first Café had been pre-transferred onto the sheets, allowing facilitators to directly refer to prior statements. At each table, the host introduced the theme, summarized input gathered at previous tables, and facilitated an open exchange on the residents’ personal associations, past experiences, and potential ideas for shared group activities. The responses were documented directly on the topic sheets. Additionally, field notes were taken. In a concluding plenary round, all documented input was reviewed and presented to the full group. 

#### 3.3.2. Findings

The analysis of the second Matters of the Heart Café generated narrative summaries for each of the twelve heart topics. These summaries highlight participants’ personal associations, past experiences, and ideas for potential group activities.

1. Holidays and Travels: Participants shared diverse holiday and travel experiences, ranging from trips within Germany and neighbouring countries (Italy, Austria, France) to other European destinations (Spain, England, Scotland, Scandinavia) and even further abroad (USA, China, Africa). One participant vividly recalled a safari in Kenya with encounters with elephants and the Massai. Others highlighted simpler holidays in the garden or on short bus trips. Activities such as skiing, hiking, and sailing were frequently mentioned, alongside memories of family holidays with parents, grandparents, or children. Several expressed a wish to share travel stories and learn about each other’s favourite destinations.
2. Cooking and Baking: Many participants described their enjoyment of cooking and baking, ranging from making jams, soups, and traditional dishes to preparing and sharing fruit desserts, cakes, and bread. Baking was often emphasized, with mentions of cookies, apple cakes, and other fruit pastries. While some noted that they did not particularly enjoy cooking, they still valued baking and hosting. Biographical references included experiences of running a household or even a farm. Several participants highlighted their interest in cooking or baking together and in exchanging knowledge about recipes.
3. Dressing up: Participants emphasized their joy in dressing up nicely, especially for church, concerts, theatre visits, or dances. Many described a preference for beautiful clothes and elegant shoes, sometimes with high heels, as well as make-up such as lipstick, mascara, rouge, or nail polish. Some recalled tailoring or sewing experiences in the family, wearing traditional clothing such as a Dirndl, or borrowing clothes from siblings. While everyday clothing was more practical, dressing up for special occasions was described as meaningful.
4. Animals: Participants reported a strong preference for animals, with dogs, cats, and horses most frequently mentioned as favourites. Several shared biographical experiences such as growing up with pets (including horses, dogs, cats, birds, rabbits, hamsters, guinea pigs, or farm animals) or encounters with animals during safaris. Some also described fear of dogs, while still appreciating animals from a distance. Across accounts, participants expressed joy in remembering their animals, watching animal films and documentaries, visiting the zoo, and wishing for animal visits in the group.
5. Sports: Participants mentioned a variety of sports interests including handball, volleyball, badminton, and skiing. Football was sometimes associated with rejection, but also with family traditions and personal enthusiasm. Some wished to talk about their favourite club, including experiences of traveling with fans to matches. Family connections to football and ice hockey (husband, children, grandchildren) were emphasized. The local football stadium emerged as both a sports venue and a landmark for walks and sightseeing. Some participants offered to explain the rules of different sports to the group.
6. Family and Friends: Participants emphasized the central importance of family, frequently mentioning children, grandchildren, siblings, or parents. They expressed pride in their families, recalled childhood responsibilities such as caring for siblings during the war, or shared biographical stories including parental influence, artistic backgrounds, and special love stories like interdenominational marriages. Friendships were described as equally meaningful, from lifelong companions to close weekly contacts, fading ties, or strong bonds with fellow residents. Several participants wished to share stories about family histories, friendships, children, or love stories.
7. Music and Dancing: Participants expressed a wide range of musical preferences, with many highlighting a fondness for folk music, Schlager, and waltzes. Classical music and jazz were also mentioned, and some named specific artists such as Marlene Dietrich. Dancing was described as a source of joy, especially waltz, polka, and foxtrot. Several participants had rich biographical experiences with music, including playing instruments such as piano, accordion, singing in choirs, or attending dance courses. Participants also voiced clear wishes for the present: they would like to listen to music, sing, or dance together in the group, and some even expressed interest in performing again, for example, by playing the accordion.
8. Religion and Church: Participants spoke about their experiences of going to church, with many describing regular attendance on Sundays or for special occasions, sometimes even walking long distances. For some, church visits were associated with dressing up, evening services, or moments of rest and peace. Accounts of prayer practices included table prayers, praying with the family, and a particular importance of the Lord’s Prayer, while others did not pray at home. Several recalled their active involvement in church life, such as holding children’s services, singing in church choirs, or to live in a convent.
9. Regional Identity and Dialects: Participants described their diverse regional and cultural backgrounds, mentioning origins from different regions of Bavaria, other German regions, and former German territories. Several highlighted regional dialects. Expressions of local identity included regional greetings, family roots in other regions or abroad, and feelings of being at home in Munich. Individual life paths were shared, such as moving for education or work. Participants also expressed an interest in knowing where the others come from and some want to teach their dialect to the group. In addition, memories of beer garden culture were highlighted, including traditional food, music, and beer mugs, along with the wish to share these customs with the group.
10. Art and Handicrafts: Participants described painting as a meaningful and enjoyable activity, closely linked to personal expression. They spoke about their favourite colours and about preferred subjects such as nature motifs (trees, rivers, forests) as well as abstract watercolour painting in bright tones. Some also reported on textile handicrafts, which were not only hobbies but also served practical purposes such as making clothes for the family. Several expressed the wish to paint together, combined with music and conversation. Others mentioned showing photos of their own artworks and contributing stories connected to them, or suggested creating collective pieces such as garden-themed paintings. One participant offered to prepare painting templates for the group.
11. Nature: Participants expressed a strong connection to nature, gardens, and hiking. Many emphasized joy in their own gardens and wished to share related experiences with the group—for example, talking about their gardens, planting flowers together, or growing herbs such as chives, parsley, and mint. Walking and hiking, both in the mountains and in the countryside, were frequently described as sources of happiness and wellbeing, with favourite destinations including the Alps, South Tyrol, and Austria. Some participants highlighted their wish to tell stories about time spent in nature or to show others aspects of the natural environment. Some talked about gardens as shared spaces of activity and relaxation.
12. Movies and Theatre: Participants frequently recalled their enjoyment of cinema visits, especially comedies and romantic films. They also spoke about their favourite actors, e.g., Heinz Rühmann and Greta Garbo. Several described a strong connection to theatre, both as audience members and through their own active involvement, such as school theatre, amateur theatre groups, or even professional training and work in the film industry. Some participants also expressed that they would like to talk about actors and films. Across different accounts, participants emphasized that they would still like to act today.

### 3.4. Care Staff Consultation

Based on the findings from Matters of the Heart Cafés 1 and 2, a care staff consultation was conducted in each facility. In two facilities, the consultations were conducted with two female staff members each, and in one facility with one male staff member. All worked in the field social care within the facility and had completed training in gerontological social care. Three of them (one in each facility) held a nursing qualification and were responsible for leading social care either at the facility level or within their respective units. All had several years of professional experience in this field and were highly familiar with the participating residents. These staff members supported the entire co-production process and also participated in the Cafés in a supportive role. Together with them, we discussed suitable peer hosts for each heart topic, particularly in cases where several participants showed strong interest in the same theme or when one individual expressed interest in multiple topics. For each theme, one or two residents with a particularly strong personal connection to the topic were selected to take on the hosting role. Efforts were made to ensure that every participant had the opportunity to act as a peer host at least once. After the final assignment of peer hosts to the topics, the sequence of sessions, their specific dates, and the corresponding preparation meetings with each peer host were discussed and jointly scheduled. The schedule was designed to align with the daily routines of the residents, the organizational structure of the care facilities, and the availability of supporting staff.

### 3.5. Preliminary Session Design

In the next step, preliminary session concepts were developed for each heart topic. It was ensured that each session concept incorporated peer-led elements, opportunities for co-creativity, and components of sensory stimulation. Three activities were developed and integrated into multiple sessions and adapted to the respective themes: sensory boxes, guided imagery, and the short game “Top or Flop.” Sensory boxes—containers prepared with objects hidden from view but accessible by touch—were used at the beginning or during sessions to stimulate tactile perception and introduce thematic materials (e.g., natural items for the theme nature and hiking). Guided imagery, often connected to the peer host’s narrative (e.g., a shared holiday trip) and sometimes enriched with sounds, images, or scents, provided short stories that served either as an introduction or as a continuous thread throughout a session. Finally, “Top or Flop” encouraged participants to collectively rate pictures, songs, films, well-known personalities, or other thematically relevant items, thereby stimulating conversation, reminiscence, and group interaction. To inform the session design, relevant literature on evidence-based and best practice approaches for the respective topics, as well as implementation methods tailored to the target group, was reviewed. Finally, drafts for twelve intervention sessions—each focusing on one heart topic—served as the basis for joint refinement, individual tailoring and final planning during the subsequent preparation meetings with the peer hosts.

### 3.6. Preparation Meetings with the Peer Hosts

Preparation meetings with the peer hosts were held a few days before each group session to allow time for adaptations and preparations based on their input while supporting the possibility of recall of the discussed content. A topic-specific guide was developed for each session based on relevant literature, while maintaining a consistent meeting structure. Each meeting began with open storytelling, followed by guided questions to elicit details and meaningful aspects of the topic. In the second part, hosts shaped their role by deciding what to share or demonstrate, which activities to co-lead, and which thematic snacks and beverages to provide. Meetings lasted approximately 30–60 min. A summary of the key content covered during each session’s preparation meeting is outlined in Appendix A (Table A1).

### 3.7. Final Session Design

In the final step, the pre-designed session drafts were adapted based on the input of the peer hosts and given meaningful titles accordingly. Key information from the preparation meetings was summarized in structured notes to guide facilitation and provide prompts to support the peer hosts during session implementation, for example in storytelling. Session materials such as pictures, maps, objects, and playlists were individualized according to the hosts’ narratives and experiences, and thematically fitting snacks and beverages were prepared as requested. This step resulted in twelve ready-to-use sessions per long-term care facility, each supported by tailored materials and designed to combine a coherent structure with flexibility for incorporating the peer hosts’ personal input.

### 3.8. Implementation of Sessions

The developed sessions were implemented in all three participating facilities within small groups of 9–10 participants who had been involved in the development process. Group sessions lasted between 60 and 90 min, were conducted between breakfast and lunch, and took place once a week over a period of 12 weeks. Weather permitting, they took place outdoors; otherwise, in the communal spaces of the facility. Each session included a short mid-break for the joint consumption of the selected snacks and beverages, with session-specific decorations prepared to create an engaging atmosphere. Sessions opened with a welcoming ritual, followed by an interactive introduction of the theme and the presentation of the peer host. Implementation was co-facilitated by the peer host and two members of the research team, with flexible support from one to two care staff depending on the host’s competencies. Each session concluded with a short closing ritual, including a personal farewell and words of appreciation for all participants, followed by a farewell song chosen individually by each group. Table 4 presents the core elements of three of the twelve session themes as they were ultimately implemented, focusing on the peer-led, multisensory, and co-creative components. Since the peer-led and corresponding multisensory components varied across facilities, only one implementation example per session is presented in the table. Appendix A (Table A2) presents the core elements of the sessions for all twelve themes.

## 4. Discussion

The present study reports on a seven-step co-creation process for developing and implementing a social health intervention for people living with dementia. This process integrated the complementary expertise of people living with dementia, care staff, and researchers. People living with dementia held sole decision-making authority in selecting the intervention themes, shared responsibility for shaping the design of individual sessions, and joint responsibility for implementing the intervention in their role as peer hosts.

The analysis of the Matters of the Heart Cafés identified twelve meaningful intervention topics. Across these topics, participants emphasized individual preferences, special interests, positive experiences, and feelings of happiness and appreciation. They also described various ways in which they could contribute to group sessions related to their heart topics, such as sharing experiences, engaging in joint activities, showing and teaching others, and telling stories. Participants further expressed a strong wish and interest to learn more about the experiences, characteristics, and preferences of their peers. Building on these findings, twelve intervention sessions were developed, each incorporating peer-led elements, opportunities for co-creativity, and components of sensory stimulation. Each session was jointly prepared, individualized, and implemented in collaboration with at least one person living with dementia serving as a peer host. 

### 4.1. The Co-Creation Process

As the findings demonstrate, the applied co-creation process was well suited to capturing the lived experiences of people living with dementia in developing the social health intervention. Nevertheless, the process and the distribution of decision-making authority still fall short of the ideal of involving people living with dementia collegially in all stages of the research process or placing them fully in control of the research question, process, and methods (Innovations in Dementia, 2023; Walter et al., 2025). Moreover, it is important to acknowledge structural factors that limited the extent to which an equal partnership between researchers and participating people living with dementia could be achieved in our approach. Although reporting participants’ MMSE scores was intended to illustrate that individuals across a wide range of cognitive abilities can be meaningfully involved when roles are adapted to their strengths, one could argue that doing so while not assessing the cognitive abilities of professional researchers introduces asymmetry. Furthermore, the research team was employed and financially compensated through their university positions, whereas the participating people living with dementia were neither contracted as employees nor financially compensated for their contribution as experts by experience—an imbalance that unfortunately remains common in the research field (Groothuijse et al., 2024). However, calls for financial compensation are becoming increasingly prominent, as such compensation acknowledges the contributions of all individuals involved (Groothuijse et al., 2024).

The ideal of conducting research on fully equal terms throughout the entire research process is, however, rarely achieved in practice (Niner et al., 2023). A wide range of barriers has been identified in the literature as contributing to this situation, some of which apply to participatory research in general, such as time limitations for essential relationship building and for the overall research process, often driven by funding and financial constraints (Bethell et al., 2018; Gaffy et al., 2022; Niner et al., 2023). In addition, specific barriers exist in co-approaches with people living with dementia, mainly related to cognitive impairments. These include (1) self-doubt about their ability to contribute, along with fears of making mistakes or feeling embarrassed (Niner et al., 2023; Waite et al., 2019); (2) researchers’ lack of confidence in their capacities while needing to produce high-quality research output (Gaffy et al., 2022); (3) gatekeepers, such as concerned caregivers, who aim to protect individuals from distress or overexertion (Bethell et al., 2018; Waite et al., 2019); and (4) ethics committee standards based on rigid assumptions about decision-making capacity (Gaffy et al., 2022; Niner et al., 2023).

In designing our co-creation process, the barriers outlined above were an important underlying consideration. Our primary concern, however, was to balance the protection of potentially vulnerable individuals with avoiding paternalistic practices and ensuring their right to meaningful involvement. We were particularly keen to prevent tokenistic or procedural “tick-box” co-creation (Gaffy et al., 2022). This rationale also guided our decision to organize those stages of the process in which people living with dementia held decision-making authority exclusively with them. In contrast, most co-creation sessions include a broader range of stakeholders (Wang et al., 2019). These mixed-group settings can pose challenges for people living with dementia, who report that their contributions are not always prioritized, that parts of the discussion feel less relevant or accessible, and that others may speak more quickly or speak over them (Lord et al., 2022). Even within the groups composed solely of people living with dementia, participants varied considerably in their functional and cognitive abilities. Thus, it was essential to ensure that all individuals could contribute in ways that aligned with their abilities. Some were able to participate in discussions in a structured manner, whereas others found it more difficult to follow the content or to express themselves. To support their participation, we actively facilitated engagement through targeted prompts, offering multiple choice options, and summarising or simplifying topics discussed by the group.

An important facilitator in our co-creation process, also noted in other studies (Bethell et al., 2018; Niner et al., 2023), was the already established and trusting relationship with people living with dementia and care staff. Another facilitating factor was that participants were not only motivated by the idea of making a difference for others or future residents (Waite et al., 2019), but were also able to directly participate in and benefit from the intervention they had co-created.

### 4.2. The Matter of the Heart Cafés

The World Café method inspired the Matters of the Heart Café 1 and guided the structure of the Matters of the Heart Café 2. As a participatory method with a flexible format, its design principles (The World Café Community Foundation, 2025) align well with key recommendations for co-creation with people living with dementia (Wang et al., 2019), making it an excellent approach for generating qualitative data within co-creation processes (Löhr et al., 2020). Its successful use with this population has also been demonstrated by Keogh et al. (2021), who applied a Policy Café to involve people living with dementia and carers. Our experiences are consistent with theirs, suggesting that the method can be recommended for future co-creation projects involving people living with dementia, because it can be adapted to different abilities and purposes. In addition, Agnello et al. (2025) identified 248 co-creation methods in their scoping review on co-creation in public health research, many of which could likely be adapted for use with this population. Future studies should evaluate different participation methods for people living with dementia and develop recommendations regarding their suitability for specific research aims, dementia stages, and support needs, as well as provide detailed best practice examples.

The identified heart topics closely align with what previous studies describe as meaningful activities for people living with dementia (e.g., Tournier et al., 2023). They ranged from past interests, such as traveling, to activities that remain part of everyday life in the care facility, such as spending time in the garden or watching films. Most topics can be mapped onto the six life areas of meaningful activity proposed by Tuijt et al. (2020): physical activity, looking after one’s household, enjoyable and leisure activities, hobbies and personal interests, staying mentally active, and social activities and community involvement. The questions in the first Matters of the Heart Café, which covered not only meaningful activities but also meaningful events, situations, stories, and themes, broadened the thematic range and brought more identity related topics to light (Tierney & Beattie, 2020). This was particularly evident in themes such as regional identity and dialects.

Moreover, the second Matters of the Heart Café encouraged participants to share extraordinary memories and personal stories, such as special journeys to meet the Maasai or unique love stories. These can be described as nostalgic recollections, referring to momentous and atypical life events (Ismail et al., 2022). Evoking nostalgia is rooted in, but goes beyond, reminiscence therapy, which is a well-established and effective nonpharmacological intervention in dementia care (Y. Han et al., 2025; Redulla, 2020), but typically does not specify which types of memories should be recalled. In contrast, nostalgia based approaches highlight memories that are particularly meaningful or extraordinary (Ismail et al., 2018). Although dementia related neurological changes may reduce the effectiveness of nostalgia based interventions by limiting access to autobiographical memory (Dodd et al., 2022), an increasing number of studies have examined its use among people living with dementia and have shown positive findings regarding feasibility and benefit (Dodd et al., 2022; Ismail et al., 2022; Oliver et al., 2024). Nostalgia appears especially promising in social health interventions in this population, as it fosters social connectedness by creating emotional bridges between the self and others (Ismail et al., 2018).

Shifting the focus from oneself to what one can offer to the group, and vice versa, further strengthened the component of social connectedness, emphasized reciprocity, and represents perhaps the most novel aspect of the findings from the Matters of the Heart Cafés: People living with dementia mentioned many specific ways they could actively contribute to group situations and expressed strong interest in getting to know others better and engaging in shared activities. These findings stand in contrast to common practice in residential care, where interaction in group activities is often limited (Gebhard et al., 2024) and participants are frequently assigned a passive, consuming role (Theurer et al., 2015). This highlights the untapped potential of people living with dementia as active contributors to social life in long-term care and opens new avenues for promoting social health.

### 4.3. The Social Health Intervention

The co-creation process resulted in an intervention that has the potential to foster empowerment and enable people living with dementia to engage in meaningful social interaction by sharing what matters to them, learning about others through joint activities, and discovering shared experiences and interests. Although residents in long-term care live together, share spaces, and follow similar routines (Gebhard & Frank, 2024; Stöhr et al., 2022), meaningful social interactions rarely develop naturally. Many people living with dementia report that existing social opportunities do not meet their needs (Casey et al., 2016; Chapman et al., 2024), and some cannot name a single meaningful occasion to connect (Gebhard et al., 2025). In addition, many consciously avoid contact with fellow residents, perceiving them as unsuitable social partners, often due to assumed lack of shared interests (Chapman et al., 2024; Gebhard et al., 2025). The social encounters that do occur are frequently described as superficial, limited to polite greetings, and associated with feelings of disconnection (Abbott & Pachucki, 2017; Chapman et al., 2024; Gebhard et al., 2025). At the same time, people living with dementia spend very little time with family and friends from outside the facility (Gebhard et al., 2024; Weimer et al., 2022), and ongoing staff shortages further restrict opportunities for social interaction with care staff (Boamah et al., 2021). These factors underscore the importance of drawing on the readily available social resource of fellow residents. Whether our developed intervention was effective in facilitating relationships between residents was evaluated using social network analysis, focusing on changes in the structure and quality of relationships within the participant group (Robins et al., 2023; Scott & Carrington, 2011). In addition, we conducted descriptive profile analyses to identify which participants benefited most from the intervention and whether cognitive functioning in particular contributed to greater benefit. The results on the intervention’s effectiveness are reported elsewhere (Gebhard & Ellinger, 2025).

A key feature of the intervention that deserves particular emphasis is the active involvement of peer hosts. Each participant was enabled to take on this role in ways that reflected their preferences, abilities, and wishes. This builds on the pioneering work of Skrajner et al. (2014), who demonstrated that people living with dementia can lead group activities, yet extends it by focusing on highly personal themes, with each participant hosting a session on their own “heart topic.” This also responds to Tournier et al.’s (2023) call to provide opportunities and support for people living with dementia to lead or co-lead activities. However, implementation was not without challenges, as it required flexibility and improvisation, particularly in balancing authenticity in the peer host role with maintaining a positive group experience. Depending on their functional and cognitive abilities, participants enacted the role in diverse ways: some took on leadership from the start—welcoming the group, sharing prepared reflections, or dressing to match their theme—while others contributed more selectively or asked the research team to narrate parts of their story, in which case we aimed to preserve agency by seeking confirmation and clarification. Despite these challenges, we strongly recommend integrating this approach into intervention practice and research. Future studies should examine the added value of peer-led compared with professionally led interventions and identify factors that facilitate successful implementation.

### 4.4. Limitations

The limitations of this study relate to several areas: methodology, evaluation, manuscript preparation, and practical application. 

The methodological limitations correspond closely to the key challenges of involving people living with dementia in research (Wang et al., 2019). Thus, the generalizability of the findings is strongly limited for three main reasons: (1) The relatively small convenience sample was drawn exclusively from one federal state in southern Germany and consisted predominantly of women, which may not reflect the broader population of people living with dementia across different care models, regions, or gender identities. In addition, no data were collected on further participant characteristics such as education, cultural background, dementia type, behavioural and psychological symptoms of dementia, or emotional state—factors that substantially shape social relationships—further limiting the contextualization of the findings. Future studies would therefore benefit from a deliberate sampling strategy aimed at structural representativeness, weighted according to the distribution of key characteristics in the population (e.g., age, education, gender, dementia type, cultural background). (2) We excluded people who were cared for in bed, those with young onset dementia, and those who were not verbally communicative to a minimal degree, as the study procedures required some level of verbal engagement. This further limits the transferability of the findings to more diverse groups of people living with dementia with differing abilities. Beyond representativeness, future research should also consider the heterogeneity within this group—including personal characteristics and dementia-related factors—in a more differentiated manner, both for co-creation processes and for social health interventions. (3) Moreover, participants identified and elaborated on topics that were personally meaningful to them, without the intention of representing all people living with dementia.

Further, as in most co-creation research in health settings, no detailed evaluation of the co-creation process itself was conducted (Slattery et al., 2020). One of the few examples on which future co-creation processes can build is the study by Bloska et al. (2024), which systematically evaluated the experiences of people living with dementia involved in an international study together with their informal caregivers. The evaluation focused on four themes: (1) expectations for involvement, (2) perceived contributions to the research study, (3) benefits and challenges, and (4) recommendations for future dementia research. Another limitation is that we missed the opportunity to co-write this paper together with the participating people living with dementia. For future studies, we recommend interweaving the manuscript preparation process with the co-creation process itself and allocating dedicated time for this purpose within the overall project timeline. Finally, the challenges inherent in residential long-term care, such as understaffing, limited training opportunities for care staff, and insufficient institutional funding, pose substantial barriers to translating this research into clinical practice.

## 5. Conclusions

The present study makes a relevant contribution to an emerging field of intervention research for people living with dementia: the promotion of social health. It fills an evidence-based intervention scaffolding with the lived experiences of people living with dementia through a co-creation process. By presenting the development process and the implemented program in detail, this study enhances the transferability and applicability of its approach. The findings call on researchers to embrace co-creation as a means of developing tailored and meaningful social health interventions for people living with dementia. Moreover, our work aims to encourage researchers and practitioners to take bolder steps in reimagining the social world of residential long-term care by giving people living with dementia active, visible, and meaningful social roles. In doing so, we take one step toward the “social revolution in residential care” that Theurer et al. (2015) called for almost a decade ago.

## Figures and Tables

**Table 1:** Sample characteristics.

Variables	M ± SD	Min–Max
Age (years)	85.79 ± 4.81	77–93
Functional status (care level)	2.93 ± 0.75	2–4
Cognitive status (MMSE)	18.07 ± 4.31	10–25

Note. M = Mean, SD = Standard Deviation, MMSE = Mini-Mental State Examination.

**Table 2:** Themes and questions for the Matters of the Heart Café 1.

Themes	Questions
Me for Myself	What brings you joy or fun? (In the past/today)
	What makes you happy? (In the past/today)
	Is there something you would like to (re)try or do (again)?
Me for the Group	Is there something you are particularly good at? Would you like to show or teach it to the group?
	Is there a topic you know a lot about? Would you like to share your knowledge with the group?
	Is there a story or experience you would like to share with the group? For example, what are you especially proud of or what has been your most beautiful experience?
The Group for Me	What interests you about the other people in the group?
	What would you like to learn from others?
	What would you like to do together with others?

**Table 3:** Summary of results from Matters of the Heart Café 1.

Topic	Me for Myself	Me for the Group	The Group for Me
Holidays and Travels	Joy in traveling and hiking; preferences include holidays at the sea and in the mountains, especially in the Alps, but also in the local region	Sharing stories with the group about holidays, experiences while traveling alone as a woman, longer stays abroad, and special travel destinations such as the Orient	To hear from others about their holidays, and travel destinations; to engage in conversations about where the others have been on vacation
Cooking and Baking	Joy in cooking and baking, specifically in making jam, Christmas pastries, preparing meals, and enjoying food; preference for traditional German dishes	Cooking jam for everyone and preparing fruit dishes; sharing and enjoying food together	To learn whether others can cook with vegetables; to prepare fresh food together
Dressing up	Joy in dressing up nicely and getting ready for special occasions	/	/
Animals	Preference for animals and watching animal documentaries and films; positive experience of keeping pets; joy in animal visits, going to the zoo; interest in monkeys	Telling others about animals, especially horses	/
Sports	Joy in outdoor sports, e.g., hiking, walking, cycling, and swimming; joy in supporting the favourite football team; positive experiences in supporting children in sports	Share stories about football and the favourite team; showing cycling skills; shared activities such as going for walks	/
Family and Friends	Joy in the presence of family and friends, special importance placed on family still being there; happiness in visits from children and grandchildren; happiness when people connect with each other; appreciation of friendships with other residents, importance of having someone to talk to	Sharing family stories with the group, including the number of children and what has become of them; share personal stories on grandchildren, parents, family life history, and love	Wishing to know from others whether they are aware that they are liked; wishing to find out whether the others would also like to meet regularly; wondering whether the others know me
Music and Dancing	Joy in concerts, dancing (especially waltz, polka, and foxtrot), and listening to music; preference for folk music, Schlager, Polka, and classical music; positive experience playing instruments (piano, accordion)	Singing together, making music together, listening to folk music together	Listening to music and singing together, especially folk music; interest in learning what songs others like
Religion and Church	Joy in singing church songs, praying the Lord’s Prayer; positive experience working in the church, holding children’s services	Teaching the group church songs and prayers; telling the others that our world is beautiful and good	/
Regional Identity and Dialects	Joy in going to a traditional beer garden, drinking beer or shandy	Telling the group about the own home regions; sharing the own dialect with the group; teaching the group what belongs to a traditional beer garden visit	Learn more about where the others come from; in the context of flight from war and displacement, where they resettled; wishing to know how the others feel about Munich
Arts and Handicrafts	Joy in painting, specifically watercolour nature scenes or abstract motifs, and in textile handicrafts, especially knitting, embroidery, and sewing	Showing photos of own artwork, sharing self-embroidered pieces; engaging in joint handicrafts, painting nature scenes together	Painting together
Nature	Joy in spending time outdoors and in the garden, working in the garden, tending to a small allotment and sunbathing	Showing flowers; spending time together outdoors, nature as a shared experience	Everything together in the nature
Movies and Theatre	Joy in going to the opera, theatre, and cinema, watching TV; preference for comedies, romance films, animal films, music shows, and specific actors; positive experience in the film industry	Performing for others; sharing experiences from working in the film industry	/
General Statements	Fun is the most important thing; joy in working together on something; joy of helping others	Talk with others; showing curiosity about others; sharing what has been learned; reading aloud, such as a poem or text	Learn more about how the others are doing, what they enjoy, what they have learned/experienced, how men behaved in their lives; talk about shared experiences; spend time together; to be read to by others

**Table 4:** Core elements of three intervention sessions.

Session	Peer-Lead Elements	Co-Creative Elements	Multisensory Elements
Travels Near and Far	(1) Storytelling about safari experiences, animals, people, and traditional clothing—embedded in a tailored guided imagery; (2) describing a typical holiday day; (3) showing travel photos and souvenirs	(1) “Packing the suitcase” together with prepared items; (2) creating a group map with all travel destinations (each person adds their own photo); (3) language quiz on how to say “hello” in different common holiday languages	(A) Plane sounds, drum rhythms, animal noises, holiday music (e.g., Italian songs); (O) sunscreen; (G) tropical fruits; (V) pictures of the Big Five, Maasai, safari truck, tents, and other holiday destinations (sea, mountains); (T, barefoot) sand, small pool, shells
Loved Ones and Good Times	(1) Reading the preselected poem about the importance of friendship; (2) storytelling about one’s own love story between a Catholic woman and a Protestant man and what it meant at that time	(1) Creating a shared “Tree of the Heart” including all people and beautiful moments important to each group member; (2) exchanging compliment cards from a prepared selection or self-written ones	(A) Songs about friendship; (G) “Merci” chocolates, cookies, juice; (V) charms, small pictures of positive things and group members, “tree of hearts,” compliment cards; (T) creative crafting activities
Stories on Stage and Screen	(1) Storytelling about own experiences as a theatre actress; (2) performing a prepared sketch; (3) teaching the group how to express emotions such as joy, anger, or fear through acting	(1) “Top or Flop” of famous actors; (2) quiz on famous film music; (3) acting out emotions together; (4) jointly developing an improvisational theatre scene performed live by staff	(A) Movie music; (G) prosecco and salmon sandwiches; (V) stage set for the sketch, movie posters, pictures of actors, and facial expressions of emotions

Note. Auditory (A), olfactory (O), gustatory (G), visual (V), tactile (T).

## Data Availability

The datasets generated and analysed during the current study (German-language transcripts and fieldnotes) are not publicly available due to the sensitive nature of the qualitative data. Transcripts and fieldnotes contain personal narratives that may include potentially identifying information. Data may be available from the first author on reasonable request.

## Abbreviations

The following abbreviations are used in this manuscript:

A	auditory
G	gustatory
INTERDEM	Early detection and timely INTERvention in DEMentia
M	Mean
MMSE	Mini-Mental State Examination
O	olfactory
SD	Standard Deviation
T	tactile
V	visual

## Appendix A

**Table A1:** Key contents of the preparation meetings with the peer hosts.

Topic	Key Contents
Holidays and Travels	Ask the peer host about their travel experiences and favourite destinations. Inquire about travel companions, means of transport, luggage, traditions, and funny stories. Talk about souvenirs or photos they still have, and bring a selection of suitable pictures for them to choose from.
Cooking and Baking	Ask the peer host about their cooking and baking experiences, favourite cakes/dishes, and the utensils they used. Find out who helped them in the kitchen and for which occasions they cooked or baked. Let them choose a recipe to bake together (prepare a small selection).
Dressing up	Ask about the styles and fashion trends in their lives, and their favourite clothes. Find out which types of beauty products, hairstyles, makeup, shoes, and accessories they particularly liked or used frequently. Ask if they would like to share a beauty routine with the group. Bring a selection of pictures of style icons and let the peer host choose which ones to bring to the session.
Animals	Ask the peer host about their experiences with animals, especially with dogs or their own pets. Talk about favourite animals and funny stories. Ask about possible tricks with dogs and what they would like to show during the session. Meet with the therapy dog and the dog handler 30 min before the session to rehearse the planned commands and tricks.
Sports	Ask about their favorite sport and their passion as a fan. Talk about favourite athletes and memorable experiences related to them. Let the peer host explain sports rules or traditions. Bring a selection of sport games and let the peer host decide which one to do together during the session.
Family and Friends	Ask the peer host about their family, friends, and loved ones. Find out about their parents, spouses, children, or grandchildren, as well as family trips, special memories, and their love story. Talk about friendships and what makes a good friend. Bring several poems about friendship and family and let them choose one. Bring a selection of compliments and positive pictures and let them select a few.
Music and Dancing	Ask about memories and stories related to special songs, favourite singers, genres, and concert experiences. Play a selection of songs while showing pictures of the performers, and ask which ones they like best to create a personal playlist. Bring a small selection of illustrated dance step guides (e.g., waltz, polka) and practice the chosen dance together to demonstrate and teach it to the group.
Religion and Church	Ask about their religious experiences, traditions, and festivals. Find out which religions they know and how they celebrated events such as their baptism or wedding. Bring a selection of prayers and let them choose one to read to the group. Ask about religious symbols and songs and whether they would like to present one. Explore what their faith means to them.
Regional Identity and Dialects	Ask about their home region, traditions, and what is typical of their place of origin. Bring a selection of dialect words and let the peer hosts choose their favourites or contribute their own. Find out about local dishes, costumes, music, proverbs, and common stereotypes. Ask what they could teach to others from their traditions and which story they would like to share.
Art and Handicrafts	Ask about their own creativity and artworks, and whether they have pieces to show. Ask if they would like to demonstrate or guide the creation of an artwork in the group. Find out about their knowledge of famous paintings and artists. Let them choose favourite artworks or colouring templates from a selection.
Nature	Ask the peer host about their hiking and nature experiences, favourite routes, companions, duration, and animals or plants they have seen. Prepare a selection of natural materials, sounds, and hiking songs, and let the peer hosts choose. Ask about their backpacks and clothing. Bring drawing templates of mountains, trees, alpine flowers, and mountain animals, and let the peer hosts decide which to use.
Movies and Theatre	Ask about their experiences and stories related to theatre and cinema, including their favourite plays and films. Find out how they prepared for a theatre visit and who accompanied them. Talk about actors and any personal acting experiences. Bring a selection of short sketches, let them choose one, and practice it together to perform for the group.

**Table A2:** Core elements of the 12 intervention sessions.

Session	Peer-Lead Elements	Co-Creative Elements	Multisensory Elements
Travels Near and Far	(1) Storytelling about safari experiences, animals, people, and traditional clothing—embedded in a tailored guided imagery; (2) describing a typical holiday day; (3) showing travel photos and souvenirs	(1) “Packing the suitcase” together with prepared items; (2) creating a group map with all travel destinations (each person adds their own photo); (3) language quiz on how to say “hello” in different common holiday languages	(A) Plane sounds, drum rhythms, animal noises, holiday music (e.g., Italian songs); (O) sunscreen; (G) tropical fruits; (V) pictures of the Big Five, Maasai, safari truck, tents, and other holiday destinations (sea, mountains); (T, barefoot) sand, small pool, shells
Kitchen Stories	(1) Storytelling about the egg liqueur cake as the favourite family birthday cake; (2) giving advice on the recipe preparation and guiding the correct sequence in the baking puzzle	(1) Baking puzzle (egg liqueur cake recipe divided into picture cards to be arranged in the correct order); (2) baking the cake together—each person is responsible for one step; (3) eating the cake	(O) Ingredients and freshly baked cake; (G) egg liqueur cake and coffee; (V) pictures of various cakes and dishes; (T) sensory boxes with baking utensils and ingredients for the egg liqueur cake, hands-on baking experience
Fashion and Dressing up	(1) Demonstrating how to create an updo hairstyle; (2) advising other participants on individual suitable colours and styles using a selection of prepared images of different dirndls	(1) “Top or Flop” with style icons; (2) creating a joint birthday party outfit—embedded in a tailored guided imagery	(A) Music of famous style icons (e.g., Marilyn Monroe); (O) perfume and cosmetics; (G) sparkling wine and cookies; (V) pictures of style icons; (T) sensory boxes with different fabrics, beauty products, and accessories
Time with Animals	(1) Describing the beloved dog “Wasti”; (2) demonstrating pre-practiced commands and tricks with the therapy dog together with the dog handler	(1) Work with the dog together in the group; (2) “Top or Flop” of cutest animals	(A) Animal show songs and dog barking sounds; (G) cookies shaped like zoo animals; (V) pictures of farm animals, zoo animals, and pets, observing the dog; (T) petting the dog, sensory boxes with a dog bowl, leash, and toys
The World of Sports	(1) Storytelling, embedded in a tailored guided imagery, about being a fan of football club 1860 Munich and traveling with the fan club to matches; (2) explaining the “offside” rule	(1) “Top or Flop” of sports legends; (2) playing bowling together	(A) Fan chants and football songs; (G) typical stadium food (grilled sausages, beer); (V) pictures of famous athletes and various stadiums; (T) balls from different sports
Loved Ones and Good Times	(1) Reading the preselected poem about the importance of friendship; (2) storytelling about one’s own love story between a Catholic woman and a Protestant man and what it meant at that time	(1) Creating a shared “Tree of the Heart” including all people and beautiful moments important to each group member; (2) exchanging compliment cards from a prepared selection or self-written ones	(A) Songs about friendship; (G) “Merci” chocolates, cookies, juice; (V) charms, small pictures of positive things and group members, “tree of hearts,” compliment cards; (T) creative crafting activities
Our Playlist	(1) Introducing the selected favourite song and storytelling about its personal significance; (2) demonstrating the waltz dance steps	(1) Quiz about instrument sounds; (2) creating a group playlist; (3) singing along to the peer host’s selected songs (lyrics provided); (4) dancing the waltz steps together	(A) Sounds of various instruments, selected songs, Viennese waltz music; (G) sparkling wine and berries; (V) pictures of singers and bands related to the selected songs
Faith and Religion	(1) Storytelling about growing up in a monastery and leading children’s services; (2) reading the preselected prayer	(1) Quiz about world religions; (2) singing and praying together; (3) designing a large gratitude candle—each person adds a wax decoration and writes what they are grateful for	(A) Ecclesiastical music, church bells, organ sounds, and religious songs; (O) incense, candle wax; (G) berries and red juice; (V) pictures of world religions, famous churches, and religious symbols; (T) candle wax
Where We Come from	(1) Storytelling about one’s own homeland—embedded in a tailored guided imagery; (2) demonstrating how to cut radish and shape pretzels; (3) explaining the meaning and origin of one’s favourite dialect words	(1) Beer tasting in the group (guessing different beer types); (2) creating a group map showing where everyone comes from and which dialect is spoken there; (3) quiz on dialect words and how to say “I love you” in different dialects	(A) Traditional brass band and folk music; (O) freshly baked pretzels; (G) various beer types, pretzels, radish; (V) traditional clothing, beer garden decorations, pictures of regional dishes; (T) different beer mugs
Art and Handicrafts	(1) Storytelling about being an art teacher; (2) showing pictures of own paintings and artwork; (3) teaching the group painting techniques for creating a group artwork; (4) explaining how to mix colours	(1) Creating a group painting; (2) analysing famous artworks	(O) Acrylic paints; (G) red wine and berries; (V) pictures of paintings by artists such as Picasso, Monet, and Munch; (T) canvas, paintbrushes
In Nature on Foot	(1) Storytelling about personal hiking experiences, favourite routes, mountain storms, and observed animals—embedded in a tailored guided imagery; (2) reciting a humorous hiking poem	(1) Creating a group collage of a mountain landscape using colouring templates and natural materials	(A) Hiking songs, nature sounds of wind, water, storms, cowbells, and birds; (O) natural materials such as pine branches, bark, and moss; (G) bread, bacon, cheese, and wheat beer; (V) pictures of mountains, peak crosses, cows, marmots, and typical backpack items; (T) sensory boxes with natural materials
Stories on Stage and Screen	(1) Storytelling about own experiences as a theatre actress; (2) performing a prepared sketch; (3) teaching the group how to express emotions such as joy, anger, or fear through acting	(1) “Top or Flop” of famous actors; (2) quiz on famous film music; (3) acting out emotions together; (4) jointly developing an improvisational theatre scene performed live by staff	(A) Movie music; (G) prosecco and salmon sandwiches; (V) stage set for the sketch, movie posters, pictures of actors, and facial expressions of emotions

Note. Auditory (A), olfactory (O), gustatory (G), visual (V), tactile (T).
